# 依库珠单抗治疗溶血性阵发性睡眠性血红蛋白尿症的中国真实世界研究

**DOI:** 10.3760/cma.j.cn121090-20231106-00250

**Published:** 2024-02

**Authors:** 乐宇 王, 青林 胡, 苗 陈, 辰 杨, 冰 韩

**Affiliations:** 中国医学科学院、北京协和医学院北京协和医院血液科，协和转化医学中心，北京 100005 Department of Hematology, Peking Union Medical College Hospital, Chinese Academy of Medical Science and Peking Union Medical College, Beijing 100730, China

**Keywords:** 阵发性睡眠性血红蛋白尿症, 溶血性, 依库珠单抗, 疗效, 安全性, Paroxysmal nocturnal hemoglobinuria, Hemolysis, Eculizumab, Efficacy, Safety

## Abstract

**目的:**

观察依库珠单抗在中国治疗溶血性阵发性睡眠性血红蛋白尿症（PNH）患者中的有效性和安全性。

**方法:**

回顾性分析2022年12月至2023年7月接受至少3个月足量依库珠单抗治疗并随访至少3个月的溶血性PNH患者资料，评估依库珠单抗治疗1、2、3和6个月后临床及实验室指标的变化，并记录突破性溶血（BTH）、血管外溶血（EVH）的比例及不良反应发生情况。

**结果:**

共纳入9例PNH患者，其中男6例，女3例，中位年龄54（28～69）岁。5例患者为经典型PNH，4例为PNH/再生障碍性贫血（AA）患者。在使用依库珠单抗前，血红蛋白尿发作次数为每月5（1～25）次。4例患者在接受依库珠单抗前需要输血，5例合并血栓，1例有肾功能损害。依库珠单抗的治疗中位时间为6（3～7）个月，随访时间为治疗后3（3～6）个月。使用依库珠单抗后，血红蛋白尿发作次数为0（0～1）次。在随访期内，无新发血栓形成。治疗后各个时间节点的LDH均较基线明显下降；部分患者HGB较基线有上升；输血依赖者均摆脱输血。疲劳功能评分（FACIT-Fatigue评分）在治疗后平均提升17.3分。2例患者发生BTH，经对症好转。3例出现了轻度不良事件，无严重不良事件及死亡。

**结论:**

依库珠单抗对中国溶血性PNH的相关症状有较好的控制作用，在一定程度上能减少输血，改善患者生活质量，且安全性较好。

## Abstract (en)

**Objective:**

To evaluate the efficacy and safety of eculizumab in the treatment of paroxysmal nocturnal hemoglobinuria (PNH) in China.

**Methods:**

Data from PNH patients who received at least 3 months of full-dose eculizumab and were followed for at least 3 months between December 2022 and July 2023 were retrospectively collected. We evaluated changes in clinical and laboratory parameters after 1, 2, 3, and 6 months of eculizumab treatment. The rates of breakthrough hemolysis (BTH), extravascular hemolysis (EVH), and the occurrence of adverse reactions were also monitored.

**Results:**

The study included nine patients, six males and three females, with a median age of 54 (28–69) years. 5 of the patients had classic PNH, while 4 had PNH/AA. The number of episodes of hemoglobinuria was 5 (1–25) per month before eculizumab. 4 patients required blood transfusion, 5 had thrombosis and one had renal impairment before eculizumab. The median time to eculizumab was 6 (3–7) months and the followup period was 3 (3–6) months after treatment. The number of episodes of hemoglobinuria following eculizumab was 0 (0–1). During the followup period, no additional thrombotic events occurred. LDH at any time after eculizumab was lower than at baseline, and some patients' HGB increased. All transfused patients became transfusion-independent after receiving eculizumab. The FACIT-Fatigue score improved by an average of 17.3 points following treatment. 2 patients developed BTH and improved with symptomatic treatment. There were three adverse events that caused mild symptoms. There are no serious adverse events or deaths.

**Conclusion:**

Eculizumab can effectively control the hemolytic-related symptoms of PNH in China, reducing the need for blood transfusions to some extent, while also demonstrating a higher safety profile.

阵发性睡眠性血红蛋白尿症（PNH）是一种起源于造血干细胞的PIG-A基因突变导致的获得性造血干细胞克隆性疾病。血管内溶血是其主要的且突出的临床特征。发作性或慢性血管内溶血会导致患者出现贫血、血红蛋白尿及其他相关并发症，如血栓形成、腹痛、吞咽困难和勃起功能障碍等，严重影响生活质量甚至寿命[1]–[3]。依库珠单抗是一种人源化单克隆抗体，于2007年在美国获批上市，可以阻断末端补体C5活化，抑制了血管内溶血，并进一步减轻了血栓形成、肾功能损害、平滑肌功能障碍等溶血相关的并发症，极大地改善了PNH患者的生活质量，并使PNH患者的寿命提升到接近正常人水平[4]–[5]。上市16年来，已有大量的国外临床试验、真实世界的研究证实其疗效及安全性[6]–[8]。直到2022年底，依库珠单抗才在中国上市，但目前能够使用标准剂量的患者较少，国内依库珠单抗治疗PNH的真实世界数据相对匮乏。在我们中心登记的PNH患者中，有部分接受了依库珠单抗治疗并进行了规律随访，本研究对这些患者的临床数据进行了回顾性分析。

## 病例与方法

一、病例资料

本研究为回顾性队列研究，以2022年12月至2023年7月在北京协和医院诊断PNH且规律使用依库珠单抗至少3个月的患者为研究对象。PNH的诊断和分类参照国际PNH兴趣小组（IPIG）标准[5],[9]，分为经典型、伴有骨髓衰竭（BMF）的PNH（PNH/BMF）和亚临床型。纳入标准：①年龄≥12岁；②明确诊断PNH经典型或PNH/BMF；③使用依库珠单抗前有血管内溶血的临床表现或并发症，且患者乳酸脱氢酶（LDH）≥1.5正常上限（ULN）；④足量使用依库珠单抗至少3个月；⑤有完整的病历资料者。

二、研究方法

1. 临床资料收集：包括人口学信息、病史（包括血栓形成史、输血次数及输血量）、体格检查、基线实验室检查结果［包括HGB、WBC、ANC、网织红细胞计数（Ret）、LDH、总胆红素（TIBL）、血肌酐、血清铁蛋白等］、除依库珠单抗外的其他治疗情况。

患者采用依库珠单抗治疗后1、2、3和6个月，收集患者的上述临床资料及实验室检查结果。治疗前及6个月收集PNH克隆大小的数据（Flaer阴性粒细胞比例）。临床资料来自医院病历系统，或采用当地检查结果，患者通过门诊复查、电话沟通等方式进行随访，随访截至2023年9月30日。

2. 依库珠单抗治疗：所有患者在接受脑膜炎奈瑟菌疫苗接种，且至少2周后开始依库珠单抗治疗。依库珠单抗600 mg每周1次×1个月，从第2个月开始 900 mg每2周1次静脉输注。

3. 疗效判定标准及安全性分析：记录治疗后不同时间点的临床表现。记录用药后1、2、3和6个月LDH的变化（相对于基线）及≤1.5 ULN的比例；记录上述时间点患者HGB水平（较基线升高≥15 g/L认为是HGB明显上升）、输血依赖患者脱离输血依赖的比例；其他实验室数据的变化；记录突破性溶血（BTH）、血管外溶血（EVH）的比例；记录基线及随访期末疲劳功能评分（FACIT-Fatigue评分）；记录不良反应，不良反应分级参考国际通用不良反应分级（CTCAE）5.0版。

BTH：在治疗后LDH下降，溶血性贫血改善，但再次出现LDH升高，排除其他引起LDH升高的因素，且出现至少1种新的或加重的血管内溶血症状或体征［如疲劳、血红蛋白尿、腹痛、呼吸短促或呼吸困难、贫血及主要不良血管事件（MAVE）］等[10]。

EVH：使用C5抑制剂后，血管内溶血被控制，但仍然发生持续贫血、或贫血、黄疸加重、网织红细胞计数明显升高、脾大等表现，抗人球蛋白试验阳性[11]。

FACIT-Fatigue评分：得分范围为0～52，得分越高表明疲劳有所改善[12]。

## 结果

1. 患者临床特征和治疗：共纳入9例患者，男6例，女3例，中位年龄54（28～69）岁。5例（55.6％）患者为经典型PNH，4例（44.4％）为PNH/AA。基线时HGB为（75.0±14.1）g/L、RBC（2.52±0.46）×10^12^/L、Ret（176.1±81.2）×10^9^/L、WBC（4.28±1.66）×10^9^/L、ANC（2.50±1.29）×10^9^/L、PLT（168.9±75.20）×10^9^/L、LDH（1770.9±949.8）U/L、TIBL（40.8±33.4）µmol/L、肌酐83（40～228）µmol/L、血清铁蛋白25（7～132）µg/L、PNH克隆（82.5±16.9）％。5例经典性PNH患者在使用依库珠单抗前均采用对症治疗，2例长期使用司坦唑醇，2例除输血外未接受其他治疗，1例未治疗。4例PNH/AA患者中，2例在使用依库珠单抗前分别使用环孢素A 12个月及18个月，1例使用他克莫司2年，1例使用司坦唑醇3年，其中2例间断输血。这些患者从使用依库珠单抗至随访期末，原有的其他治疗没有改变。

所有患者在使用依库珠单抗前，均存在血红蛋白尿发作，每月中位发作次数为5（1～25）次。4例患者在接受依库珠单抗前6个月需要输血，每年中位输血量为3（1～10）个单位；5例合并血栓，部位分别为下肢深静脉血栓和门脉血栓、右髂静脉血栓和腹部血栓、脑梗死、腹部血栓、腹部血栓；1例有肾功能损害，CKD 3级。

确诊至使用依库珠单抗的中位时间为10（3～30）年，依库珠单抗治疗中位时间为6（3～7）个月，随访时间为治疗后3（3～6）个月。

2. 疗效：所有患者在使用依库珠单抗后，血红蛋白尿发作均较基线明显改善，至随访期末的发作次数为0（0～1）次，与依库珠单抗前的每月5（1～25）次相比明显减少。在随访期内，所有患者均无新发血栓形成。

依库珠单抗治疗1、2、3及6个月后，实验室指标变化见表1。LDH水平相较于基线均大幅下降，HGB水平较基线略有上升。接受治疗3个月后的9例患者中，3例HGB明显升高，其中2例超过100 g/L，其余6例无明显变化；治疗6个月后的4例患者中，1例HGB明显上升，达到正常水平，其余3例无明显变化。治疗后的所有患者的LDH较基线均明显降低。患者的其他实验室指标治疗后与基线的数据见表1。使用依库珠单抗前的4例有输血需求的患者，在治疗期间均摆脱了输血依赖。1例患者在治疗前肌酐较高，治疗后其肌酐保持稳定。仅两例患者在治疗后6个月进行了PNH克隆检测，其基线及治疗后6个月时的PNH克隆分别为83.6％、88.1％，85.52％、91.4％。

**表1:** 依库珠单抗治疗不同时间点PNH患者实验室指标变化

实验室指标	基线	依库珠单抗治疗时间			
		1个月	2个月	3个月	6个月
LDH（U/L，*x±s*）	1 770.9±949.8	223.2±59.5	245.8±76.3	247.7±88.7	199.5±78.5
HGB（g/L，*x±s*）	75.0±14.1	83.3±11.8	86.8±14.0	90.1±23.7	91.3±35.1
RBC（×10^12^/L，*x±s*）	2.52±0.46	2.83±0.55	2.67±0.42	2.73±0.64	2.57±0.86
Ret（×10^9^/L，*x±s*）	176.1±81.2	195.4±78.4	234.8±131.0	196.6±91.3	241.0±169.0
PLT（×10^9^/L，*x±s*）	168.9±75.20	182.1±89.4	170.2±99.5	173.7±87.7	97.5±50.9
TBIL（µmol/L，*x±s*）	40.8±33.4	24.6±8.4	31.6±16.6	35.1±19.6	42.7±6.9
WBC（×10^9^/L，*x±s*）	4.28±1.66	3.56±1.24	3.10±1.34	3.42±1.43	3.24±2.14
ANC（×10^9^/L，*x±s*）	2.50±1.29	1.91±1.00	1.78±1.00	1.82±0.90	2.00±1.53
Cr [µmol/L，*M*（范围）]	83（40~228）	75（42~200）	76.5（40~182）	77.3（46~202）	74.8（65~95）
PNH克隆（%，*x±s*）	82.5±16.9	−	−	−	89.8±2.3

注 PNH：阵发性睡眠性血红蛋白尿症；Ret：网织红细胞绝对计数；TBIL：总胆红素；Cr：血肌酐；−：无数据

将患者分为经典型PNH和PNH/AA两组，两组基线和治疗1、2、3及6个月后的LDH水平分别为（1 543.0±1 178.1）U/L对（2 055.8±600.0）U/L、（250.0±42.7）U/L 对 （189.8±65.8） U/L、（260.8±69.3）U/L对（230.8±90.4）U/L、（256.6±88.3）U/L对（236.5±101.3） U/L、304.0 U/L对（164.7±44.4）U/L，PNH/AA组LDH水平相较于基线的下降幅度略大于经典型PNH组。

采用FACIT-Fatigue评分评价疲劳功能，结果显示在治疗前及随访末FACIT-Fatigue评分分别为（22.4±10.4）分和（39.7±9.1）分，随访终点FACIT-Fatigue评分较基线平均提升了17.3分。

3. BTH：2例患者发生BTH。发生的时间分别为使用依库珠单抗后2个月和3个月，1例患者治疗后2个月LDH正常，因使用依库珠单抗的时间延迟了1 d而发生了溶血，出现血红蛋白尿，LDH为2 299.6 U/L，立即注射依库珠单抗，而后溶血停止，LDH恢复正常。另1例患者在用药期间因上呼吸道感染，发生1次溶血，出现明显血红蛋白尿，LDH 1 780 U/L，在感染好转后，血红蛋白尿停止，后续LDH仍维持正常。

4. EVH：本研究中的确存在LDH水平快速下降而Ret增高、HGB未能升高或恢复的患者，但因为我们未进行治疗后红细胞补体C3测定或Coombs试验检测，所以不能准确判定是否存在EVH。

5. 安全性：3例患者出现了不良事件：1例轻度头痛，1例关节痛、脚部轻微肿胀，1例上呼吸道感染，发热1 d，经对症治疗均好转。无严重不良事件及死亡。

## 讨论

依库珠单抗已在我国可及，尚缺乏我国大陆人群的大宗病例的报道。本研究是我国大陆迄今为止首个也是最大例数的单中心报道。我们的研究结果显示，依库珠单抗治疗后，所有PNH患者的溶血均迅速和持续减少，溶血发作频率大幅下降，在6个月的随访期内仅报告了2次溶血。基线有输血需求的患者均摆脱了输血依赖，无新发血栓形成。

血管内溶血是PNH的一个突出特征，也是患者发生严重临床后果的核心[1]–[2]。LDH是溶血的生化标志物。本研究的9例患者在使用依库珠单抗1个月后，LDH从正常范围上限的7倍左右，降低至正常水平或略高于正常水平，且后续2、3和6个月均保持稳定。TRIUMPH研究中，PNH患者在接受依库珠单抗治疗1周后LDH水平从正常范围的10倍左右降至正常或略高于正常水平，后续保持稳定[6]。日本的AEGIS试验结果显示，与基线相比，在治疗1周后平均LDH水平降低了62.8％，后续的持续依库珠单抗治疗使LDH从基线时的正常范围的7倍左右降至正常或略高于正常水平[13]。我们的研究和其他国外的研究结果一致[7],[14]–[16]。国外报道的LDH的显著降低在用药后1周左右，我们未收集到用药后1周患者的临床数据，但用药后1个月LDH水平已基本降至正常或略高于正常水平。

我们观察到所有患者HGB一直保持稳定，略显示出上升趋势。日本的一个研究有类似发现，在早期的临床试验中未观察到HGB的显著上升[13]，而这种变化在后续的长期监测中才显现出来[17]。我们的研究已显示出依库珠单抗治疗输血需求的显著减少，在用药1个月后，4例基线有输血需求的PNH患者均摆脱了输血依赖，后续5个月均未输血。日本的PMS研究显示有近50％的患者在使用依库珠单抗治疗1年后摆脱了输血依赖[17]。韩国的一项研究报道的脱离输血率在治疗的前6个月内达到53.3％，在治疗36个月后达到90.9％[16]。欧美的早期临床试验显示在6个月（TRIUMPH）和12个月（SHEPHERD）的研究期内，约有51％的PNH患者实现脱离输血[6]–[7]。

血栓栓塞是PNH死亡的主要原因，约占40％[2]–[3],[18]–[19]，仅靠抗凝治疗通常不足以预防进一步的血栓并发症[6],[20]。Hillmen等[8]观察到长期依库珠单抗治疗的血栓栓塞发生率降低了81.8％。本研究中，使用依库珠单抗前，5例患者曾有血栓事件发生；使用依库珠单抗后，未发生新的血栓形成事件。这与文献[6],[8]发现类似。

疲劳是PNH患者最常报告的症状，可能对生活质量产生不利影响[21]。生活质量的受损不仅与贫血相关，还有慢性和急性溶血发作和游离血红蛋白清除一氧化氮的影响[1],[22]。我们的研究结果发现，依库珠单抗治疗后FACIT-Fatigue评分较基线提升了17.3分（增加>3分通常被认为具有临床意义[23]）。无论患者在使用依库珠单抗之前的输血需求和溶血水平如何，所有患者均观察到疲劳程度的显著改善。这与其他的研究结果一致，如有研究显示依库珠单抗治疗能显著提升PNH患者FACIT-Fatigue评分，改善患者生活质量[6]–[7]。

在长期治疗期间，11％～27％的患者使用依库珠单抗时可能会出现BTH[24]–[26]。我们的研究显示，在依库珠单抗用药期间，有2例（22.2％）患者发生了BTH，发作短暂轻微，发生在延迟用药1 d后和病毒性感冒时。Kulasekararaj等[10]的一项研究中报告了5例PNH患者使用依库珠单抗后发生了7次BTH事件，4次与C5抑制不足相关，2次与感染相关，1次原因不明。我们的研究结果与其类似。

虽然依库珠单抗可有效预防由膜攻击复合物介导的C5依赖性血管内溶血，但存活的PNH红细胞被C3片段调理，并通过肝脏和脾脏，在血管外被破坏[27]。在大多数接受C5抑制剂治疗的PNH患者中可观察到EVH，并导致红细胞半衰期缩短[27]–[28]。尽管接受了C5抑制剂治疗，EVH仍可表现为持续性贫血，并可能导致需要持续输血，这也可能是部分接受依库珠单抗治疗获益不佳的一个原因[27]–[29]。我们的研究也存在LDH水平快速下降而网织红细胞计数增高、HGB未能升高或恢复的患者，但因为我们未进行治疗后红细胞补体C3测定或抗人球蛋白试验，所以不能准确判定是否存在EVH。我们后续将在随访中完善这些检查。有研究显示C3补体抑制剂、D因子抑制剂、B因子抑制剂等在有效控制血管内溶血的同时，也能防止EVH[30]–[32]。但需要更多研究来证明其临床获益。

研究期间无严重不良事件及死亡发生。3例患者出现了不良事件，分别为轻度头痛，关节痛、脚部轻微肿胀，上呼吸道感染，不能明确是否与药物相关。Hillmen等[6],[14]的两项研究报道最常见的不良反应包括头痛、鼻咽炎、背痛、恶心和关节疼痛等；Brodsky等[7]的研究报道最常见的不良事件是头痛、鼻咽炎和上呼吸道感染；Kanakura等[13]和Ninomiya等[14]也报道了头痛、溶血和鼻咽炎等常见的不良事件。我们的研究结果与上述研究类似。最常见的头痛的严重程度多为轻度或中度，持续时间较短，使用非阿片类镇痛药可缓解[7]。

国外研究提示，依库珠单抗在经典型PNH中的疗效类似于或略优PNH/AA[16],[33]。由于我们的病例数过少，无法进行统计学分析，但本研究中，无论是经典PNH，还是PNH/AA，依库珠单抗均有效且安全。

尽管为回顾性研究，例数较少，随访时间较短，我们依然可以看出，依库珠单抗可以明显改善PNH患者的血管内溶血及相关的症状，降低LDH，使输血患者摆脱输血依赖，且不良反应轻微，但在短时间内对于HGB水平提升略不明显。同时由于随访时间有限，我们没有见到更多的BTH，且因检测试验的缺失也没有发现EVH。但随着随访时间的延长和检查的完善，这些问题可能在依库珠单抗使用一段时间后出现。未来还需要在更大人群及更长的随访时间内观察，才能得出确切结论。
